# Respiratory syncytial virus infection‐induced mucus secretion by down‐regulation of miR‐34b/c‐5p expression in airway epithelial cells

**DOI:** 10.1111/jcmm.15845

**Published:** 2020-09-16

**Authors:** Xizi Du, Yu Yang, Gelei Xiao, Ming Yang, Lin Yuan, Ling Qin, Ruoxi He, Leyuan Wang, Mengping Wu, ShuangYan Wu, Juntao Feng, Yang Xiang, Xiangping Qu, Huijun Liu, Xiaoqun Qin, Chi Liu

**Affiliations:** ^1^ Department of Respiratory Medicine National Clinical Research Center for Respiratory Diseases Xiangya Hospital Central South University Changsha China; ^2^ Department of Physiology School of Basic Medicine Science Central South University Changsha China; ^3^ Basic and Clinical Research Laboratory of Major Respiratory Diseases Central South University Changsha China; ^4^ Department of Neurosurgery Xiangya Hospital Central South University Changsha China; ^5^ Centre for Asthma and Respiratory Disease School of Biomedical Sciences and Pharmacy Faculty of Health and Medicine University of Newcastle and Hunter Medical Research Institute Callaghan NSW Australia; ^6^ School of Basic Medical Sciences & Academy of Medical Science Zhengzhou University Zhengzhou China; ^7^ Research Center of China‐Africa Infectious Diseases Xiangya School of Medicine Central South University Changsha China

**Keywords:** airway epithelial cells, miRNA, mucus secretion, pathway and process enrichment analysis, respiratory syncytial virus

## Abstract

Severe RSV infection is the main cause of hospitalization to children under the age of five. The regulation of miRNAs on the severity of RSV infection is unclear. The aim of the study was to identify the critical differential expression miRNAs (DE miRNAs) that can regulate the pathological response in RSV‐infected airway epithelial cells. In this study, miRNA and mRNA chips of RSV‐infected airway epithelia from Gene Expression Omnibus (GEO) were screened and analysed, separately. DE miRNAs‐targeted genes were performed for further pathway and process enrichment analysis. DE miRNA‐targeted gene functional network was constructed on the basis of miRNA‐mRNA interaction. The screened critical miRNA was also investigated by bioinformatics analysis. Then, RSV‐infected human bronchial epithelial cells (HBECs) were constructed to verify the expression of the DE miRNAs. Finally, specific synthetic DE miRNAs mimics were used to confirm the effect of DE miRNAs on the RSV‐infected HBECs. 45 DE miRNAs were identified from GEO62306 dataset. Our results showed that hsa‐mir‐34b‐5p and hsa‐mir‐34c‐5p decreased significantly in HBECs after RSV infection. Consistent with the biometric analysis, hsa‐mir‐34b/c‐5p is involved in the regulation of mucin expression gene MUC5AC. In RSV‐infected HBECs, the inducement of MUC5AC production by decreased hsa‐mir‐34b/c‐5p was partly mediated through activation of c‐Jun. These findings provide new insights into the mechanism of mucus obstruction after RSV infection and represent valuable targets for RSV infection and airway obstruction treatment.

## 1 INTRODUCTION

Respiratory syncytial virus (RSV) infection in early childhood has been associated with the development of asthma. It is worth noting that severe early RSV infection trigger an increased susceptibility of asthma and the exacerbations in most of children with asthma, which caused substantial public health cost and economical cost.^1^, ^2^ Although epidemiologic studies have revealed that over 95% children have been infected with RSV before 2 years old,^3^ severe RSV infection only occurs in a small number of RSV‐infected children.^4^ Given the significant connections between RSV infection response and the development and exacerbation of asthma, accumulating studies have demonstrated that the differential antiviral responses in asthmatics may explain these connections.^5^

The immune response and clinical manifestations after RSV infection vary greatly among children after RSV infection.^6^, ^7^ Moreover, airway obstruction is one of the main clinical manifestations of hospitalization for severe RSV infection.^8^ Compared with healthy children, asthmatic children have lung function impairment during childhood which persists into adulthood. Moreover, airway remodelling is already present in the early stage of childhood asthma.^9^ The lungs of infants with severe RSV infection also showed that desquamated epithelial cells and inflammatory cell could cause airway obstruction.^10^ It is also of particular concern that increased mucus secretion has shown to be related to the severity of RSV infection. Moreover, RSV infection could increase the composition of airway epithelial cells that secrete MUC5AC.^11^ Then, the excessive mucus secretion and reduced mucus clearance by ciliated cells lead to mucus deposition, airway obstruction, further infection and even increased bacterial colonization.^12^ However, the inner mechanism of airway mucus secretion after RSV infection is far from clear.

Interestingly, a series of recent evidences demonstrated that RSV infection regulates epithelial genes expression through epigenetic mechanisms.^13^ Epigenetic mechanisms such as DNA methylation, histone modifications and microRNAs expression could regulate transcription activities of target genes without alterations of nucleotide sequence. It has been demonstrated that aberrant miRNA expression plays a critical role in promoting the development and progression of RSV infection.^14^ miRNAs were also screened to serve as a therapeutic and prognostic factor for RSV infections.^15^, ^16^

miRNA is a type of noncoding RNA between 22 and 24 nucleotide (nt).^17^ Acting as a negative regulator of gene expression, miRNAs are predicted to target 60% of all human protein‐coding genes and involved in many biological processes, including proliferative responses and inflammatory/immune responses.^18^ Several studies have investigated the altered miRNA in RSV‐infected airway epithelial cells and the role of the altered miRNAs has recently been highlighted.^14^, ^16^, ^19^ However, the specific role of the differential miRNAs in the pathogenesis between mild RSV infection and severe RSV infection has not been fully understood.

Here, we analysed differential expression miRNAs (DE miRNAs) between healthy individuals, mildly RSV‐infected patients and severely RSV‐infected patients by screening miRNA profiling from public datasets. Then, pathway enrichment analysis, and miRNA‐mRNA function regulated network were constructed to further uncover the function of the DE miRNAs. Bioinformatics analysis screened out that hsa‐mir‐34b‐5p and hsa‐mir‐34c‐5p were negatively correlated with the severity of RSV infection. RSV‐infected airway epithelial cells further confirmed that RSV infection decreased the expression of hsa‐mir‐34b/c‐5p, which further induced mucus secretion through the activation of c‐Jun/AP‐1. Together, these results explored a new possible mechanism for mucus secretion after RSV infection in airway epithelial cells which further provided a potential target for the prevention and treatment of asthma.

## 2 MATERIALS AND METHODS

### 2.1 Search strategy

A systematic searching strategy was constructed to identify miRNA and mRNA expression profiles in public platform. Firstly, a comprehensive search was performed in GEO (www.ncbi.nlm.nih.gov/geo/) and Array Express (www.ebi.ac.uk/arrayexpress), separately. The search string used such terms as "microRNA" AND "microarray", "RSV" OR "respiratory syncytial virus" OR " respiratory infection". Secondly, datasets‐relevant studies and their reference lists were further reviewed to ensure no potential researches have been missed. Datasets were excluded according to the following criteria: (a) only cell lines were used; (b) non‐human organism; (c) non‐respiratory tissue.

### 2.2 Identification of DE miRNAs

GSE62306 was finally selected for analysis according to the above standards. Using the GEO2R program (https://www.ncbi.nlm.nih.gov/geo/geo2r/), normalization and identification of DE miRNAs from GSE62306 dataset were performed. Raw data were normalized, and the overall characteristics of value distributions and samples which were not median‐centred values were excluded. At this stage, the nasal mucosal samples from healthy children were assigned to ‘control group’. Severe RSV infection samples were assigned to 'severe group' according to grade information. The DE miRNAs were calculated by limma package built‐in R software and adjusted for multiple test by the Benjamini and Hochberg (BH) method, with the threshold criterion of adjusted *P*‐value < 0.05.^20^ Results of included miRNA expression were ranked by adjusted *P*‐values.

### 2.3 Pathway and process enrichment analysis

Pathway and process enrichment analysis were performed to determine the biological significance of DE miRNAs. By the use of Gene Ontology (GO) analysis built‐in Funrich software (http://funrich.org/index.html), DE miRNAs were statistically enriched to GO function terms. The DIANA (http://diana.imis.athena‐innovation.gr/DianaTools/index.php) webserver was used to perform Kyoto Encyclopedia of Genes and Genomes (KEGG) pathway analysis for DE miRNAs by three independent databases (TargetScan, micro T‐CDS, Tarbase). The results of GO and KEGG pathway enrichment were sorted by *P*‐value, and *P*‐value less than 0.05 was considered to be significantly enriched.

### 2.4 miRNA–target regulatory functional network construction

To identify a more valuable miRNA–mRNA interactions network, we used DIANA‐MR‐microT database (http://diana.imis.athenainnovation), which utilize target prediction algorithm, to identify potential targets of DE miRNAs. Then, the Venn plot was used to select genes overlapped in DE miRNA‐targeted genes, and DE mRNAs were identified in the included mRNA‐seq datasets. Similar to DE miRNAs identification, using the GEO2R program (https://www.ncbi.nlm.nih.gov/geo/geo2r/), normalization and identification of DE mRNAs were performed. DE mRNAs were calculated by the Benjamini and Hochberg (BH) method, with the threshold criterion of adjusted *P*‐value < 0.05. Functional networks were constructed by Cytoscape 3.7.4 through the importation of DE miRNAs and their predicted target mRNAs.

### 2.5 Cell culture

Normal primary human bronchial epithelial cells (HBECs) were purchased from Lifeline Cell Technology (Frederick, MD, USA), collected from normal adults without diagnosed lung‐related diseases with an approved protocol. HBECs were cultivated as described previously.^21^ The cells were maintained in a Ham's F12/DMEM (1:1) with the addition of transferrin (5 mg/mL), insulin (5 mg/mL), cholera toxin (10 ng/mL), epidermal growth factor (10 ng/mL), dexamethasone (0.1 mmol/L), bovine hypothalamus extract (15 mg/mL), BSA (0.5 mg/mL) and all‐transretinoic acid (30 nmol/L), at 5% CO_2_, 37°C.

### 2.6 Transfection of miRNA mimics, inhibitor and RSV infection

Synthetic hsa‐miR‐34b/c‐5p mimics (50 nmol/L) or negative control (RiboBIO, China) were transfected into HBECs following transfection protocol (R10034.5, RiboBIO, China).^22^, ^23^ 48 hours after transfection, cells were stimulated with respiratory syncytial virus strain A2 (RSV‐A2) at MOI of 3 and incubated for 24 hours. To block JNK, HBECs were cultured with SP600125 (5 μmol/L) (Selleck Chemicals, USA) for 24 hours. Then, cells were lysed in TRIzol reagent (Thermo Fisher Scientific, USA) or RIPA with 1 mmol/L PMSF, 1 mmol/L DTT and proteinase inhibitors for total RNA and protein extraction, respectively.

### 2.7 Cell viability assays

Cell viability was measured by Cell Counting Kit‐8 (Dojindo, China), HBECs were seeded in a 96‐wells plate with 10^5^ cells in 100 μL per well. After adhesion, HBECs were transfected with miRNA mimic and infected with RSV‐A2 as above. Then, cells were treated following the CCK‐8 kit protocol. The cell density at 450 nm was determined by the use of microplate reader (Bio‐rad, USA).

### 2.8 RT‐qPCR for c‐Jun, c‐Fos, MUC5AC and MUC5B

Total RNA was prepared from HBECs and quantified on a SmartSpec™ Plus spectrophotometer (Bio‐rad, USA). RT‐PCR was conducted according to the PrimeScript™ RT Master Mix Kit (Takara, Japan). Quantitative PCR (qPCR) was performed on a CFX96 Touch™ Deep Well Real‐Time PCR Detection System (Bio‐rad, USA) by the use of TB Green^®^ Premix Ex Taq (Takara, Japan) with thermal cycling conditions.^24^ Primer sequences were described in Supplementary Table 1.

**Table 1:** KEGG analysis of DE miRNAs (*P*‐value < 0.05). Enriched pathways overlapped in three independent miRNA databases (TargetScan, micro T‐CDS, Tarbase) were represented with *P*‐value and included miRNAs

KEGG pathway	TargetScan		micro T‐CDS		Tarbase	
	*P*‐value	#miRNAs	*P*‐value	#miRNAs	*P*‐value	#miRNAs
Fatty acid biosynthesis	<1e‐325	2	<1e‐325	2	<1e‐325	9
Prion diseases	0.00	1	0.000000000001108558	1	<1e‐325	6
ECM‐receptor interaction	0.02128755	7	<1e‐325	6	<1e‐325	13
Fatty acid metabolism	0.000000000136	3	0.000015083	3	<1e‐325	11
Signalling pathways regulating pluripotency of stem cells	0.00000000000285	7	0.00000000002612888	13	0.000411612	7
Mucin‐type O‐Glycan biosynthesis	<1e‐325	9	<1e‐325	12	0.01888892	5
Glycosphingolipid biosynthesis ‐ lacto and neolacto series	0.00000000000285	5	0.000007216141	6		
TGF‐beta signalling pathway			0.002174542	6	<1e‐325	14
Prion diseases			0.000000000001108558	1	<1e‐325	27
Hippo signalling pathway			0.01527432	7	<1e‐325	19
Glioma			0.001038763	6	<1e‐325	20
MAPK signalling pathway			0.00009811219	4	0.000411612	7
Transcriptional misregulation in cancer			0.04981134	4	0.003115843	8

### 2.9 miRNA RT‐qPCR

Total miRNAs was prepared with TRIzol reagent (Thermo Fisher Scientific, USA) and Phenol‐Chloroform extraction from HBECs and quantified on a SmartSpec™ Plus spectrophotometer (Bio‐rad, USA).^25^ cDNA was reverse transcribed by use of the miRcute miRNA cDNA kit (Tiangen Biotech, China), and RT products were then detected by the miRcute Plus miRNA qPCR Kit (Tiangen Biotech, China). U6 was used as an internal reference. The thermal cycling conditions were the following: 95˚C for 5 minutes, followed by 40 cycles at 95˚C for 15 seconds, 60˚C for 30 seconds and 72˚C for 20 seconds. The relative expression was calculated with the 2‐ΔΔCt method. The miRNA primer sequences were described in Table S1.

### 2.10 Western blot

Protein extraction from HBECs was performed according to previous procedures.^26^ In brief, 50 µg protein was isolated and separated from HBECs by 10% SDS‐PAGE and transferred to a polyvinylidene fluoride (PVDF) membrane. Then, the PVDF membrane was incubated with primary antibody for 12 hours and next incubated with Horseradish Peroxidase (HRP) conjugated secondary antibody. Expressions of c‐Jun (Santa Cruz, sc‐74543) and phosphorylated c‐Jun (Santa Cruz, sc‐822) were determined with corresponding antibodies.

### 2.11 Immunocytochemistry

For immunofluorescence (IF) analysis, HBECs were plated in the 24‐well plate with cover glass, fixed with 4% paraformaldehyde permeabilized with 0.1% Triton X‐100 in PBS for 15 minutes at room temperature. After blocking with 1% BSA, the HBECs were incubated with primary antibodies to RSV‐F (Abcam, ab24011), MUC5AC (Santa Cruz, sc‐21701) or MUC5B (Santa Cruz, sc‐21768) overnight at 4°C. Subsequently, Cy3 secondary antibody (Jackson ImmunoResearch, 715‐165‐150) was used to probe the primary Ab. DAPI was used for nuclear staining.^27^ IF samples were visualized using a fluorescence microscope (Carl Zeiss MicroImaging GmbH, Göttingen, Germany). Images were captured with a digital camera (Axio‐Cam ICc3, Spectra Service, Ontario, NY, USA) and analysed with AxioVision Rel. 4.7 software (Zeiss).

### 2.12 Statistical analysis

All data were analysed with GraphPad Prism Software (version 6; San Diego, CA, USA) and presented as mean ± SEM. Statistical comparisons were made by one‐way ANOVA followed by Dunnett's post hoc test. Differences were considered statistically significant for *P < 0.05, **P < 0.01 and ***P < 0.001.

## 3 RESULTS

### 3.1 DE miRNA expression profiles in RSV‐infected children with different degree

**Figure 1:**
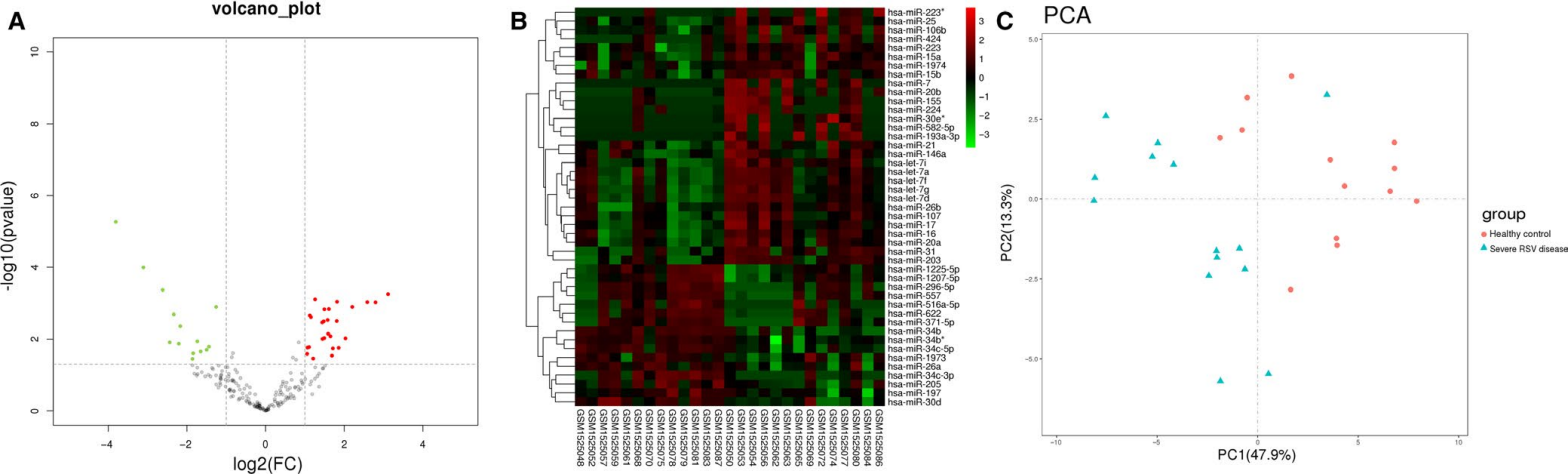
Expression profiles of miRNAs in nasal epithelial cells from control group and severe RSV‐infected group. A, Volcano plot of miRNA expression difference between healthy controls and severe RSV‐infected patients. The significantly up‐regulated miRNAs were presented as red dots and down‐regulated as green. *P* < 0.05. B, Heat map cluster showing the DE miRNAs in GSE62306. *P* < 0.05. C, A PCA plot consisting of healthy controls and severe RSV disease samples

GSE62306 was used to identify DE miRNAs in RSV‐infected children. Firstly, we analysed the DE miRNAs in all three groups provided by GSE62306 (healthy controls, mild RSV diseases and severe RSV diseases) with a total of 36 samples. By use of one‐way ANOVA, only 14 DE miRNAs were identified, and 4 of them did not show an increasing/ decreasing trend with the severity of RSV disease. Then, we performed principal component analysis (PCA) on the samples using 14 miRNAs as analysis indicators, and the results showed that the separation and classification of the three groups were not obvious (Figure S1). These results indicated that the samples in the mild group may not be heterogeneous enough to distinguish from the other two groups, which may reduce accuracy of the analysis results. Besides, the DE miRNAs in all three groups are relatively limited which may miss some of the important information and cause discrepancy in subsequent bioinformatics analysis. Thus, in our study, healthy controls group and severe RSV group were chosen for comparison with a total of 27 samples. Table S2 presented further details of included datasets. Healthy controls and severe RSV disease samples were compared. Raw data were normalized and the overall characteristics of value distributions and samples which were not median‐centred values were excluded. Finally, 27 samples including 13 control patients and 14 severe RSV‐infected patients were analysed. The distribution of value for the selected samples was shown as a box plot in Figure S2. Among all the detected 199 miRNA, 45 DE miRNAs (30 up‐regulated, 15 down‐regulated) were screened out by the adjustment of the threshold of *P*‐value < 0.05. A volcano plot and a heat map of DE miRNA were generated in Figure 1(A,B). Principal component analysis (PCA) also showed that two groups were well separated by the evaluation of these DE miRNA expression (Figure 1C).

### 3.2 Pathway enrichment analysis of DE miRNA

**Figure 2:**
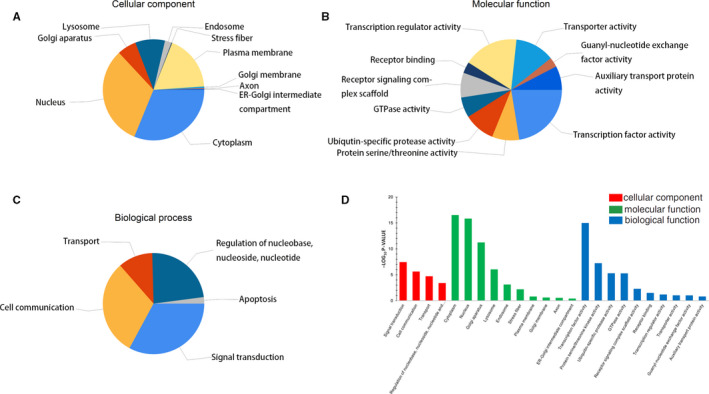
Gene Ontology classification of the DE miRNAs. A‐C, Analysis of DE miRNAs in Cellular Component, Molecular Function and Biological Process. D, The top 10 enrichment scores (−log10 *P*‐value) of enriched categories in Cellular Component, Molecular Function and Biological Process, separately

**Figure 3:**
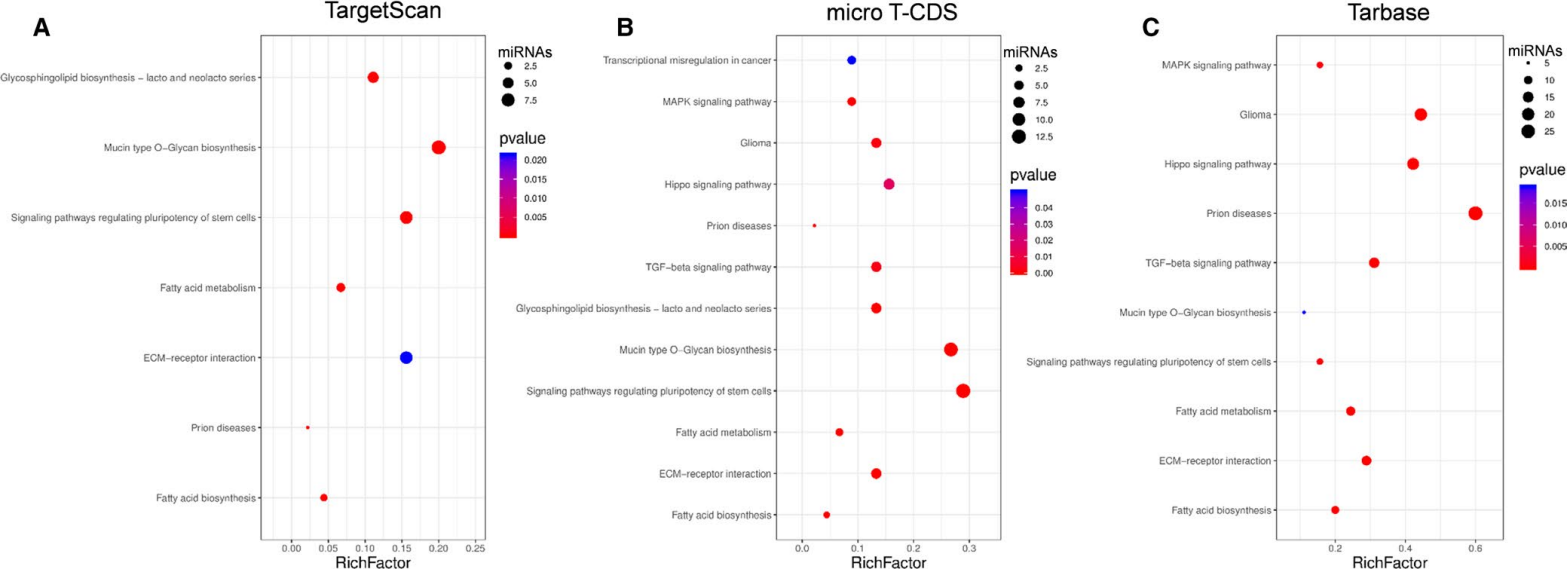
Pathways of DE miRNAs identified with KEGG pathway analysis in three databases: A, TargetScan; B, Micro T‐CDS; and C, Tarbase. Pathway name is shown on the vertical axis. Rich factor is shown on the horizontal axis. The number of candidate miRNAs in the pathway is represented by the size of the point, and the colours of the points correspond to different *P*‐value ranges

To further understand the function and pathway of these DE miRNAs, GO and KEGG analyses were performed for 45 miRNAs. Top 10 clusters with each representative enriched term were also presented, including cellular component, molecular function and biological process (Figure 2A‐D). Besides, 3 databases (TargetScan, micro T‐CDS, Tarbase) were used to analyse the KEGG pathway enrichment of DE miRNAs. In each database, *P*‐value < 0.05 was selected. Pathways enriched in more than two databases were presented in Table 1 and Figure 3. Then, 6 pathways were shown in the analysis of all the three databases, ranking in the top 20 of all the databases, including fatty acid biosynthesis, prion diseases, ECM‐receptor interaction, fatty acid metabolism, signalling pathways regulating pluripotency of stem cells and mucin‐type O‐Glycan biosynthesis.

### 3.3 DE miRNAs‐targeted gene functional network construction

**Figure 4:**
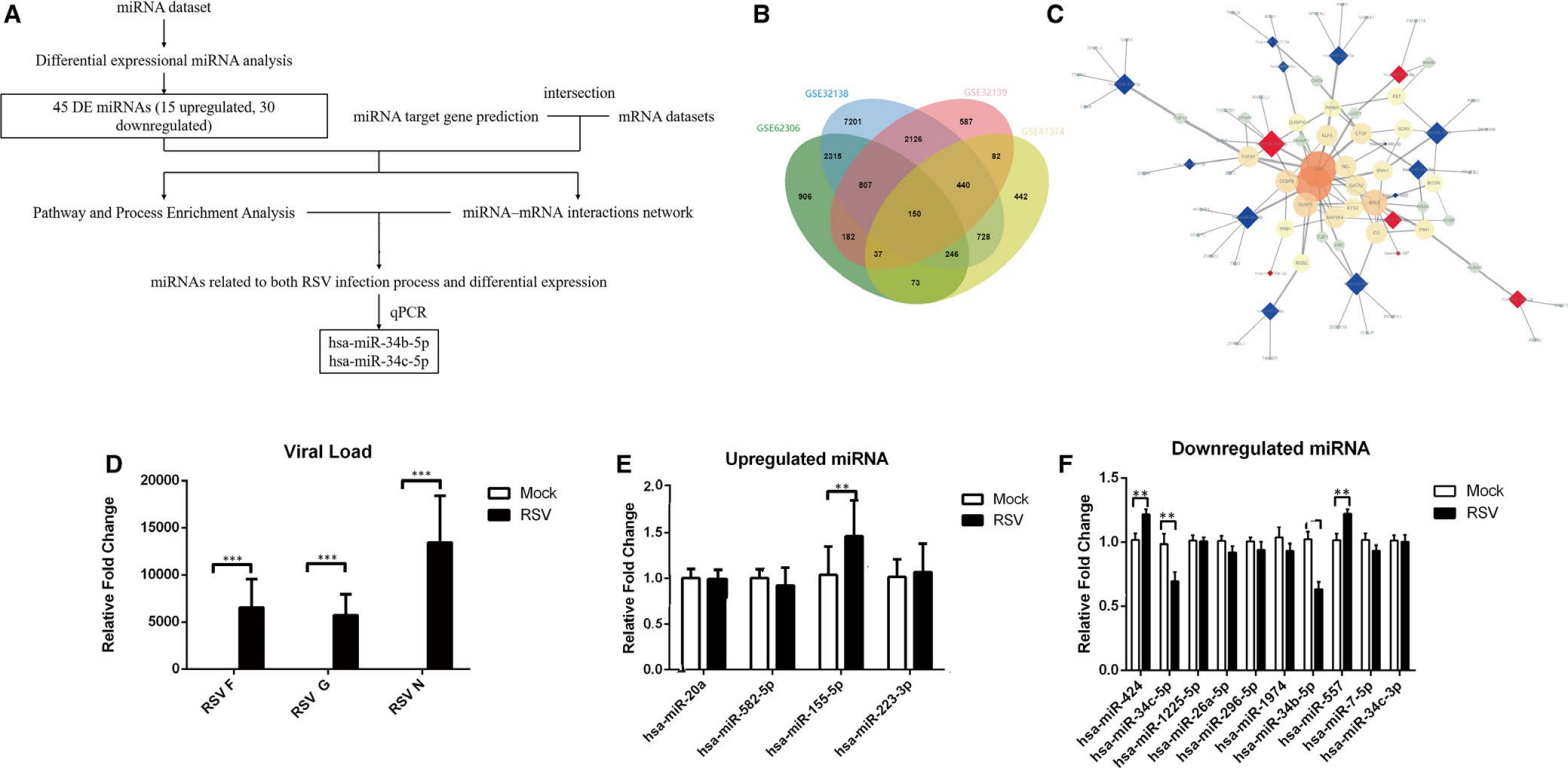
Putative interactions between DE miRNAs and target mRNAs: A, Overview of data analysis and validation strategies. B, Venn plot displays the distribution of DE miRNAs' target genes and DE mRNAs. C, Proposed networks of putative interactions between miRNAs and mRNAs after RSV infection. Regulatory networks of miRNAs and mRNAs after RSV infection were visualized with Cytoscape. Circles and quadrangles represent miRNA and genes, respectively. Blue circle nodes indicate up‐regulation, and red circle nodes indicate down‐regulation. Colour intensity denoted the level of interaction degree. Interaction degree > 3 was retained in the network. D, qPCR validation of the RSV viral load by RSV‐F, RSV‐G, RSV‐N. (E‐F). qPCR validation of the expression of DE miRNA in HBECs after RSV infection. Up‐regulated and down‐regulated miRNA in RSV‐infected HBECs (n = 6). Values are represented as mean ± SEM of at least three independent experiments. **P < 0.01, ***P < 0.001

The potential interacted gene with DE miRNAs was predicted by DIANA‐MR‐microT database. A total of 4716 genes were obtained under predict score > 0.95. To further explore the differentially expressed genes (DEGs) that may be regulated by miRNA following RSV infection, we compared the predicted target genes with the DEGs derived from RNA‐seq datasets, GSE32138, GSE32139 and GSE41374. Specific information of these three datasets was shown in Table S2. The intersection of DE miRNAs ^'^ target gene and DEGs is compared as Venn diagram in Figure 4B. There are 150 overlapping genes in the four databases. Then, we investigated DE miRNA‐target genes and protein‐protein interactions by STRING (https://string‐db.org/) and Tarbase database. DE miRNAs‐target gene network was constructed by Cytoscape 3.7.4 (Figure 4C). The network contains 18 DE miRNAs (6 up‐regulated, 12 down‐regulated) and 62 target genes (degree > 3). The most central genes in the network are JUN and FOS. They encode proteins c‐Jun and c‐Fos, which are subunits of AP‐1 transcription factor.

### 3.4 Decreased expression of hsa‐mir‐ 34b/c‐5p in RSV‐infected HBECs

We excluded 4 miRNAs with low degree (<2) in the network. The other 14 miRNAs (4 up‐regulated and 10 down‐regulated) were detected by qPCR. In RSV‐infected HBECs (Figure 4D), hsa‐mir‐155‐5p expression was up‐regulated, expression of hsa‐mir‐34b‐5p and hsa‐mir‐34c‐5p was down‐regulated. hsa‐mir‐557 and hsa‐mir‐424 show clear differences but not consistent with the trends of microarray results. (Figure 4E,F). The strategy of differentially expressed miRNA identification was showed in Figure 4A.

### 3.5 Hsa‐mir‐34b/c‐5p inhibit MUC5AC expression in RSV‐infected HBECs

**Figure 5:**
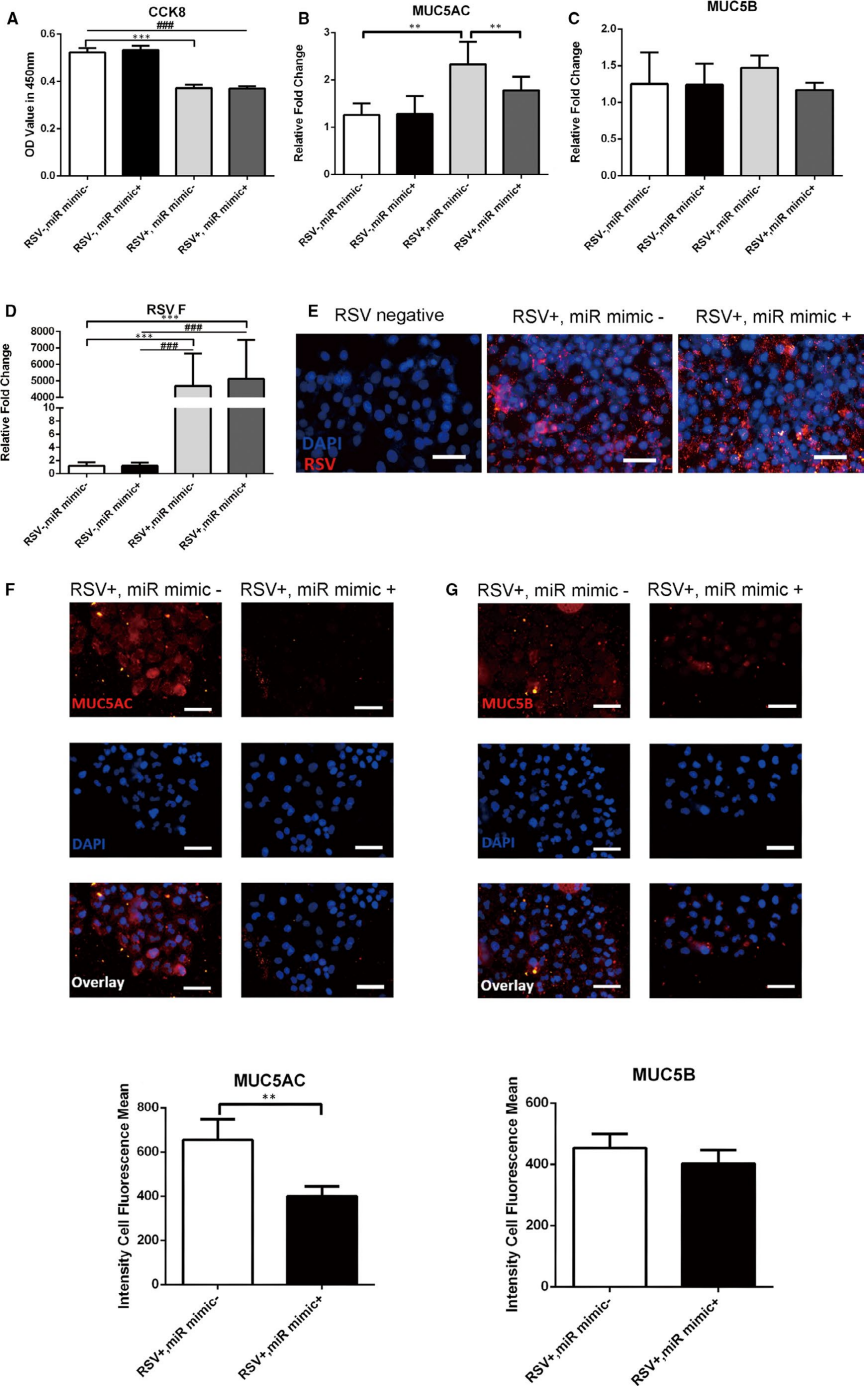
Hsa‐mir‐34b/c‐5p inhibit MUC5AC production in RSV‐infected HBECs. A, HBECs treated with hsa‐miR‐34b/c‐5p mimics (miR mimics +) or negative control mimic (miR mimics‐) were subject to CCK8 assay. B‐C, The mRNA expression of MUC5AC and MUC5B was detected by qPCR (n = 6). D‐E, Validation of the RSV‐F expression by qPCR and ICF. F‐G, The protein expression of MUC5AC and MUC5B was assessed by ICF and quantitated by the fluorescence intensity (n = 10). Scale bar, 50 μm. Values are represented as mean ± SEM of at least three independent experiments. **P < 0.01, ***P < 0.001

MUC5AC and MUC5B are major mucins that alter the composition of mucus which may exacerbate RSV infection. Our pathway enrichment analysis also showed that the pathway associated with mucin‐type O‐Glycan biosynthesis is at the top of the list. To determine the possible role of hsa‐mir‐34b‐5p and hsa‐mir‐34c‐5p in the expression of MUC5AC and MUC5B after RSV infection, hsa‐mir‐34b‐5p mimic and hsa‐mir‐34c‐5p mimic (hsa‐mir‐34b/c‐5p mimics) were pre‐transfected into HBECs before RSV infection (Figure S3). CCK‐8 assay showed that overexpression of hsa‐mir‐34b/c‐5p has no effect on the proliferation of infected HBECs (Figure 5A). After pretreatment of mir‐34b/c mimics, increased MUC5AC mRNA expression was suppressed significantly (Figure 5B). However, there was no statistical difference in the MUC5B mRNA expression (Figure 5C). Immunocytofluorescence (ICF) result also revealed that hsa‐mir‐34b/c‐5p overexpression can inhibit MUC5AC expression in RSV‐infected HBECs (Figure 5F, Figure S4A). Yet, no statistical difference in the expression of MUC5B mRNA was detected (Figure 5G, Figure S4B). In addition, by qPCR and ICF, we detected respiratory syncytial virus fusion protein (RSV‐F) in both mRNA level (Figure 5D) and protein level (Figure 5E). Results revealed that hsa‐mir‐34b/c‐5p overexpression has no significant effect on virus replication.

### 3.6 Decreased hsa‐mir‐34b/c‐5p induced MUC5AC expression through activation of c‐Jun in RSV‐infected HBECs

**Figure 6:**
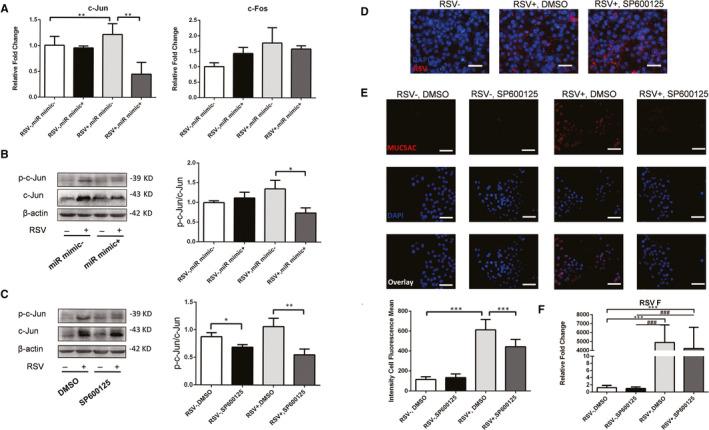
RSV infection led to increased activation of c‐Jun which was inhibited by hsa‐mir‐34b/c‐5p and SP600125. A, The mRNA expression of c‐Jun and c‐Fos was detected by qPCR (n = 6). B, HBECs were transfected with hsa‐mir‐34b/c‐5p mimics. C, HBECs were treated with SP600125. c‐Jun and p‐c‐Jun were detected by Western blot. D, Validation of the RSV‐F expression by ICF. E, The protein expression of MUC5AC was assessed by ICF and quantified by the fluorescence intensity (n = 10). Scale bar, 50 μm. F, Validation of the RSV‐F expression by qPCR. Values are represented as mean ± SEM of at least three independent experiments. **P < 0.01, ***P < 0.001

As MUC5AC expression was induced through hsa‐mir‐34b/c‐5p in RSV‐infected HBECs. We further determined the mechanism involved in the mir‐34b/c‐5p mediated MUC5AC expression. JUN and FOS, as the hub genes in DE miRNA‐gene network, were up‐regulated significantly in RSV‐infected patients.^28^ Their common transcription factor complex AP‐1 has a critical binding site of many mucins (MUC2, MUC4, MUC5AC and MUC5B),^29^, ^30^ which was activated after RSV infection.^28^ miRNA acts as a negative regulator to modulate gene expression indicating the possible correlation between the up‐regulated of hub genes and hsa‐mir‐34b/c‐5p. Consistent with our speculation, RSV infection significantly elevated c‐Jun mRNA expression which was eliminated by hsa‐mir‐34b/c‐5p mimics. However, no significant difference of c‐Fos expression was observed after hsa‐mir‐34b/c‐5p mimics overexpression (Figure 6A). Besides, the overexpression of hsa‐mir‐34b/c‐5p could inhibit the activation of c‐Jun (p‐c‐Jun) in RSV‐infected HBECs (Figure 6B) which indicated the regulation of hsa‐mir‐34b/c‐5p in AP‐1 activation. Moreover, JNK inhibitor SP600125 could also inhibit the phosphorylation of c‐Jun and MUC5AC expression in RSV‐infected HBECs (Figure 6C,E), whereas no significant change of RSV replication was observed with SP600125 existent (Figure 6D,F).

## 4 DISCUSSION

RSV is one of the most common respiratory infections in children which always shows a mild and self‐limiting upper‐respiratory illness,^31^ while about 25% RSV‐infected patients have developed into severe lower respiratory tract infections (LRTI), which led to later prevalence and exacerbation of asthma. However, the mechanism for the differential clinical manifestations and severity after RSV infection remains unclear. miRNAs have been shown to participate in modulating antiviral responses through not only the airway immune response pathways but also the virus invasion pathways.^16^, ^19^ In this study, our results demonstrated that hsa‐mir‐34b/c‐5p decreased dynamically in RSV‐infected HBECs, which was negatively related to the severity of RSV infection. Decreased hsa‐mir‐34b/c‐5p down‐regulated c‐Jun activation which further promoted the production of MUC5AC from HBECs.

By analysing miRNA microarray (GSE62306), we identified that DE miRNAs between the healthy controls and severe RSV‐infected groups. This dataset was analysed and revealed differentially expressed miRNAs in mild or severe RSV disease.^30^ Then, the results were verified by qPCR with samples of the same individuals. However, the study did not further explore the mechanism of the DE miRNAs regulating RSV infections. Here, we focused on the changes of miRNA expression in severe RSV infection and used bioinformatics analysis to predict potential regulate pathway of selected miRNAs.

Results indicated that DE miRNAs might be associated with the regulation of mucin synthesis, ECM‐receptor interaction, fatty acid metabolism and other pathological pathways which were also verified in previous studies.^32^, ^33^ It has been reported that the abnormal mucus secretion is closely related to the exacerbation of RSV infection.^10^, ^11^ Gel‐forming components of mucus covering the nasal and bronchus were mucins, containing large glycoprotein multiple O‐glycans. Although mucus could act as a protective barrier to lubricate the epithelial layer and clear foreign pathogens, the excessive accumulation of mucus may create a favourable growth environment for pathogens.^10^, ^34^ RSV‐infected epithelial cells have a tendency to differentiate into pro‐secretory phenotype, such as ciliated cell deficiency and increased number of secretory cells.^35^ This could partly restrict the spread of the virus and the invasion to susceptible cilia cells. However, severe RSV infection breaks this balance which would further amplify the pro‐secretory phenotype of airway epithelial cells.^36^ Then, the excessive mucus production and reduced ciliated cells will lead to airway occlusion, mucostasis, increased risk of further infection or even bacterial colonization.^37^

Airway epithelial cells are the primary barrier and main target site of RSV infection which play an important role in anti‐RSV response.^38^ miRNAs have been proven to regulate the immune response in RSV‐infected mammalian airway epithelial cells. Here, our results further validated that hsa‐mir‐34b/c‐5p was down‐regulated in RSV‐infected HBECs. mir‐34s is a class of highly conserved miRNA family composed of three members, miR‐34a, miR‐34b and miR‐34c.^39^ miR‐34a is encoded by its own transcript, whereas miR‐34b and miR‐34c are co‐transcribed as one gene cluster. miR‐34b/c expression is much higher than mir‐34a expression especially in lung.^40^ Interestingly, the miR‐34 family has been shown to regulate viral replication and inflammatory responses.^41^ Although mir‐34s have been used as biomarkers for certain virus infections, their specific role in RSV infection is not fully understood.

Intriguing, non‐responsive virus‐host combinations have been observed in airway secretory microRNAome in children with rhinovirus or human metapneumovirus infection.^42^, ^43^ It indicates that decreased mir‐34b/c‐5p expression in RSV‐infected HBECs is a specific virus infection response. Notably, the down‐regulation of mir‐34b/c‐5p have been also reported in the sputum and airway epithelia of asthma and COPD patients.^44^, ^45^ miR‐34/449 influence the ciliogenesis in mucociliary epithelia which is partly mediated by the post‐transcription of Cp110. mir‐34/449 deficiency in mice and frogs reduces the number and length of cilia, causing strong respiratory dysfunction.^46^ Here, we further observed that the production of MUC5AC in airway epithelial cells could be regulated by mir‐34b/c. Transcription factor AP‐1 is known to be involved in basal transcription of the MUC5AC.^28^, ^47^ The c‐Jun subunit of AP‐1, but not c‐Fos, can be inhibited by mir‐34b/c‐5p. Severe RSV infection reduces inhibition of c‐Jun, thus inducing the AP‐1 synthesis and MUC5AC production. The hypersecretion of MUC5AC increases the concentration of solids in the mucus, which makes the mucus difficult to remove.^48^ Although it has been found in previous studies that the c‐Jun NH2 terminal kinase (JNK) signalling pathway is activated after RSV infection, no modulation of JNK by mir‐34b/c was found in our RSV‐infected HBECs.^49^ These results indicate that the activation of c‐Jun is a combination of JNK pathway activation and miR‐34b/c down‐regulation.

Although this study demonstrated the critical role of decreased mir‐34b/c in RSV‐induced mucus secretion from airway epithelial cells, there are still some limitations. Indeed, we did not find a potential binding site in c‐Jun 3'UTR or 5’UTR for mir‐34b‐5p or mir‐34c‐5p although c‐Jun is negatively regulated by mir‐34b‐5p and mir‐34c‐5p partly, which indicates that the regulation of mir‐34b/c‐5p on MUC5AC may be indirect. To probe the possible downstream target genes, we constructed miRNA–target regulatory functional network (Figure 4C), which indicated that FGFR1 appears as the possible connecting molecules between hsa‐mir‐34c‐5p and c‐Jun. Besides, the prediction from TargetScan also showed that there are potential binding sites between FGFR1 and hsa‐mir‐34b/c‐5p. Moreover, the positive regulation of FGFR1 on c‐Jun has been confirmed in the previous literature.^50^ However, further work is still needed to confirm the possible target genes. Moreover, it remains unclear about other DE miRNAs or posttranscriptional mechanism in RSV‐infected HBECs. In addition, the molecular mechanism of c‐Jun activation after RSV infection remains to be further studied.

In summary, this study validated that the decreased expression of hsa‐mir‐34b/c‐5p induced MUC5AC expression in RSV‐infected HBECs. Moreover, reduced hsa‐mir‐34b/c‐5p leads to pathological production of mucin through the activation of c‐Jun which is mediated through the AP‐1 signalling pathway. These results provide some useful insights into the molecular mechanisms of mucus secretion after RSV infection and may also provide some valuable targets for RSV infection and airway obstruction treatment.

## CONFLICT OF INTEREST

The authors confirm that there are no conflicts of interest.

## AUTHOR CONTRIBUTIONS

**Xizi Du:** Conceptualization (lead); Data curation (lead); Formal analysis (lead); Investigation (lead); Methodology (lead); Project administration (lead); Software (lead); Writing‐original draft (lead); Writing‐review & editing (lead). **Yu Yang:** Formal analysis (equal). **Gelei Xiao:** Resources (equal). **Ming Yang:** Supervision (equal). **Lin Yuan:** Methodology (equal); Software (equal). **Ling Qin:** Supervision (equal). **Ruoxi He:** Resources (supporting). **Leyuan Wang:** Investigation (supporting). **Mengping Wu:** Data curation (supporting); Formal analysis (supporting). **Shuangyan Wu:** Investigation (supporting). **Juntao Feng:** Resources (supporting). **Yang Xiang:** Funding acquisition (supporting); Supervision (supporting). **Xiangping Qu:** Supervision (supporting). **Huijun Liu:** Methodology (supporting). **Xiaoqun Qin:** Funding acquisition (supporting); Supervision (supporting). **Chi Liu:** Conceptualization (supporting); Writing‐original draft (supporting); Writing‐review & editing (supporting).

## Supporting information

Fig S1

Fig S2

Fig S3

Fig S4

Table S1

Table S2

## Data Availability

All data used and analysed in this study are included in this article are available in the GEO database (https://www.ncbi.nlm.nih.gov/geo).
