# Supplementary figures and images for: Respiratory syncytial virus infection‐induced mucus secretion by down‐regulation of miR‐34b/c‐5p expression in airway epithelial cells
Source: J Cell Mol Med. 2020 Sep 16;24(21):12694–705. doi: 10.1111/jcmm.15845 (PMC7687004; doi:10.1111/jcmm.15845)

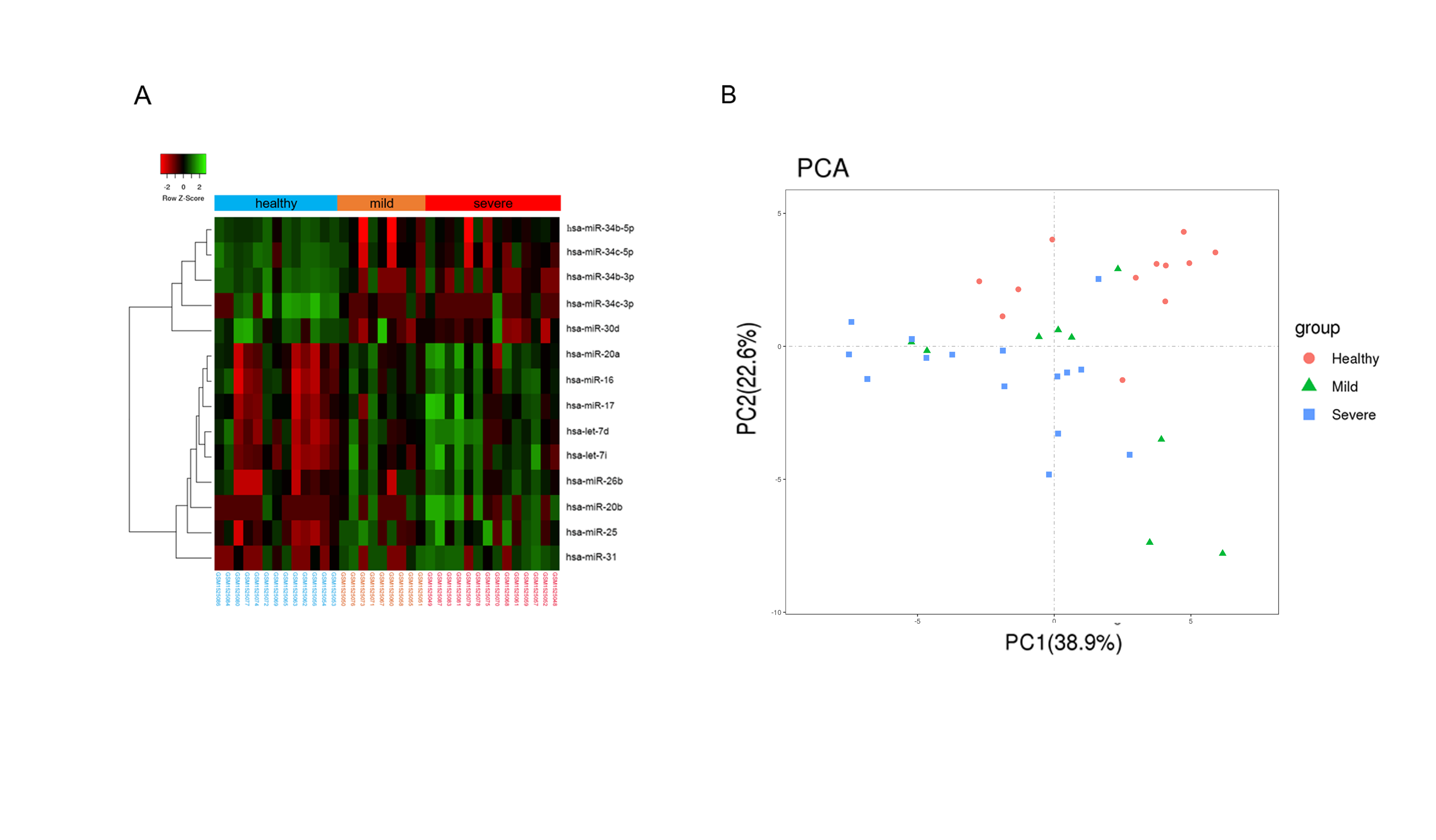

Supplement: Supplementary file 1 — Fig S1 [file JCMM-24-12694-s001.tif]

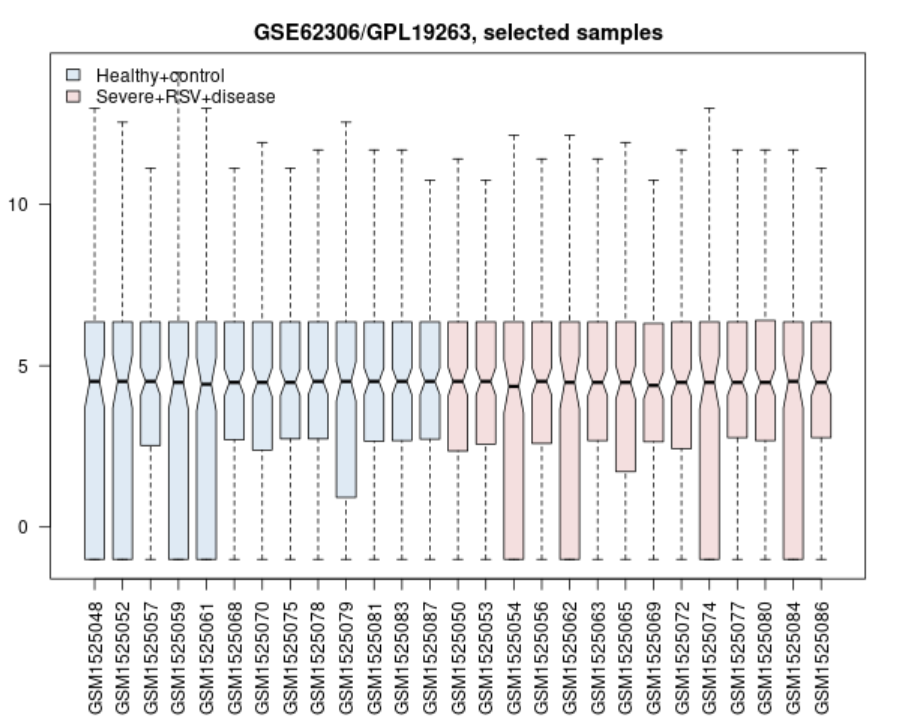

Supplement: Supplementary file 2 — Fig S2 [file JCMM-24-12694-s002.tif]

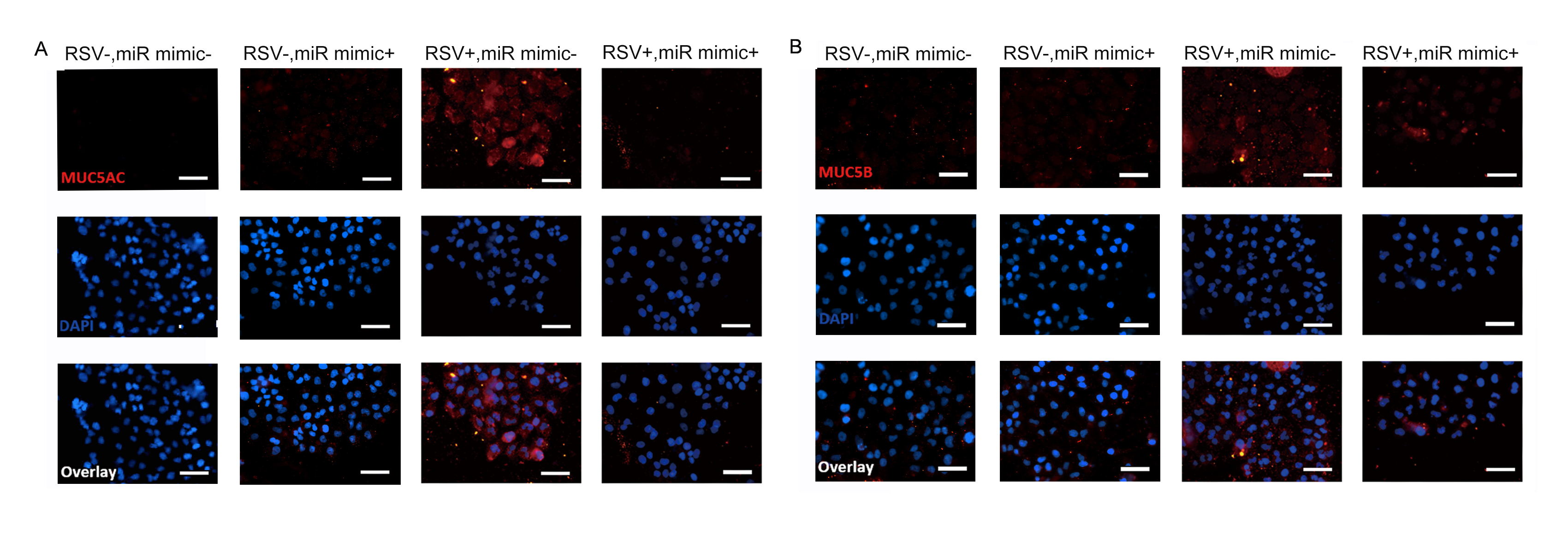

Supplement: Supplementary file 4 — Fig S4 [file JCMM-24-12694-s004.tif]
