# Supplementary material for: Respiratory syncytial virus infection‐induced mucus secretion by down‐regulation of miR‐34b/c‐5p expression in airway epithelial cells
Source: J Cell Mol Med. 2020 Sep 16;24(21):12694–705. doi: 10.1111/jcmm.15845 (PMC7687004; doi:10.1111/jcmm.15845)
Supplement: Supplementary file 5 — Table S1 [file JCMM-24-12694-s005.docx]

**Supplementary Table 1.** Primer sequence of candidate miRNAs and target genes for qPCR. U6 as a reference gene of miRNAs.

| Gene | Primer |  |
| --- | --- | --- |
| β-actin | forward | TTGCAGCTCCTTCGTTGCC |
|  | reverse | GACCCATTCCCACCATCACA |
| RSV-F | forward | CTGTGATAGARTTCCAACAAAAGAACA |
|  | reverse | AGTTACACCTGCATTAACACTAAATTCC |
| RSV-G | forward | CAACGCCAAAACAAACCACC |
|  | reverse | GGCTTGGTGGTGGTTTTCTT |
| RSV-N | forward | CAGCTGTGATTAGGAGGGCT |
|  | reverse | TCACTTGCCCTGCACCATAG |
| c-Fos | forward | CCGGGGATAGCCTCTCTTACT |
|  | reverse | CCAGGTCCGTGCAGAAGTC |
| c-Jun | forward | TCCAAGTGCCGAAAAAGGAAG |
|  | reverse | CGAGTTCTGAGCTTTCAAGGT |
| MUC5AC | forward | AGCCGGGAACCTACTACTCG |
|  | reverse | AAGTGGTCATAGGCTTCGTGC |
| MUC5B | forward | GCCCACATCTCCACCTATGAT |
|  | reverse | GCAGTTCTCGTTGTCCGTCA |
| U6 | forward | CTCGCTTCGGCAGCACA |
|  | reverse | AACGCTTCACGAATTTGCGT |
| hsa-miR-155-5p | forward | CGCGGGTTAATGCTAATCGTGATAGGGGT |
|  |  |  |
| hsa-miR-429 | forward | CGGCGGCTAATACTGTCTGGTAAAACCG |
|  |  |  |
| hsa-miR-34b-5p | forward | ACGGGCTAGGCAGTGTCATTAGCTGATTG |
|  |  |  |
| hsa-miR-34C-5p | forward | CGCGAGGCAGTGTAGTTAGCTGATTGC |
|  |  |  |
| hsa-miR-582-5p | forward | TTACAGTTGTTCAACCAGTTACT |
|  |  |  |
| hsa-miR-1225-5p | forward | GGAGTAAACAACCCTCTCC |
|  |  |  |
| hsa-miR-424 | forward | GTGCTGCAAAACGTGAGGCGCTG |
|  |  |  |
| hsa-miR-223-3p | forward | GGTGTCAGTTTGTCAAATACCCCAG |
|  |  |  |
| hsa-miR-125a-5p | forward | CGGCGGCTAATACTGTCTGGTAAAACCG |
|  |  |  |
| hsa-miR-296-5p | forward | UGUCCUAACUCCCCCCCGGGA |
|  |  |  |
| hsa-miR-557 | forward | AGACAAGGCCCACCCG |
|  |  |  |
| hsa-miR-23b | forward | AUCACAUUGCCAGGGAUUACC |
|  |  |  |
| hsa-miR-7-5p | forward | TGGAAGACTAGTGATTTTGTTGT |
