# Supplementary material for: Respiratory syncytial virus infection‐induced mucus secretion by down‐regulation of miR‐34b/c‐5p expression in airway epithelial cells
Source: J Cell Mol Med. 2020 Sep 16;24(21):12694–705. doi: 10.1111/jcmm.15845 (PMC7687004; doi:10.1111/jcmm.15845)
Supplement: Supplementary file 6 — Table S2 [file JCMM-24-12694-s006.docx]

**Supplementary** **Table 2**. Summary of public datasets GSE62306, GSE32138, GSE32139 and GSE41374.

| Data set | Microarray type | Sample type | Group | Characteristics |
| --- | --- | --- | --- | --- |
| GSE62306 | miRNA | Nasal Mucosal | Healthy (13) / mild RSV disease (13)/severe RSV disease (14) | Children |
|  |  |  |  |  |
| GSE32138 | mRNA | Human primary airway epithelial cells | Mock (4) / RSV infection (4) | - |
|  |  |  |  |  |
| GSE32139 | mRNA | Human primary airway epithelial cells | Control (4) / RSV infection (4) | - |
|  |  |  |  |  |
| GSE41374 | mRNA | Nasal wash | Healthy control (10) /RSV disease (76) | Children |
